# Prognostic Value of Vascular Calcification in Long-Term Outcomes in Obese and Non-Obese Patients with Chronic Kidney Disease

**DOI:** 10.3390/jcdd12090329

**Published:** 2025-08-28

**Authors:** Jana Uhlinova, Anne Kuudeberg, Margus Lember, Mai Ots-Rosenberg

**Affiliations:** 1 Department of Internal Medicine, Institute of Clinical Medicine, University of Tartu, 50090 Tartu, Estonia; 2 Department of Internal Medicine, Tartu University Hospital, Puusepa Str. 8, 50406 Tartu, Estonia; 3 Department of Anatomy, Institute of Pathological Anatomy and Forensic Medicine, University of Tartu, 50090 Tartu, Estonia

**Keywords:** abdominal aortic calcification, ankle–brachial index, chronic kidney disease, body mass index, long-term outcomes, obesity, vascular calcification

## Abstract

The possible protective effect of obesity in the outcomes of chronic kidney disease (CKD) patients is an understudied field. We aimed to evaluate the prognostic value of vascular calcification (VC) on long-term outcomes in obese and non-obese CKD patients. We conducted a single-centre, prospective observational study of 150 CKD patients. Patients were divided into two groups using body mass index (BMI) scores (BMI ≥ 30 kg/m^2^ and BMI < 30 kg/m^2^). Lateral lumbar X-rays (Kauppila score), the ankle–brachial index (ABI), and echocardiography were used for assessing VC. By the 11.2-year follow-up, 70 patients had died (47%). Twenty-four patients had had CV complications: stroke, myocardial infarction, decompensated heart failure, amputation caused by atherosclerosis, and aortic rupture. Among obese patients (BMI ≥ 30 kg/m^2^), only LVH was a significant predictor of CV complications (*p* = 0.01) and mortality (*p* = 0.004). In patients with BMI < 30 kg/m^2^, predictors of CV complications and mortality were ABI (*p* = 0.03; *p* = 0.009), LVH (*p* = 0.02 for CV complications) and heart valve lesions (*p* = 0.009; *p* = 0.004). There were no differences in the measured parameters of VC between the obese and non-obese groups. Moreover, no significant differences were found comparing patients with and without obesity according to the studied parameters; we found no significant differences in complications and mortality. VC in patients with CKD is a significant complication that negatively impacts outcomes. Obesity does not have a protective effect in long-term outcomes in CKD patients.

## 1. Introduction

Chronic kidney disease (CKD) is a progressive, life-threatening disease that affects more than 10% of the general population worldwide, and the most important contributor to mortality in CKD is cardiovascular diseases (CVDs) [1]. Despite the widespread opinion that the risk of complications rises from stage 3 of CKD, kidney dysfunction is an independent risk factor for CVD and death even in early stages of the disease [2]. The pathophysiology of CVD in CKD is complex, involving traditional and uraemia-related risk factors [3]. Obesity is one of the traditional risk factors for atherosclerosis and cardiovascular (CV) events in the general population and in CKD patients [4,5,6]. Moreover, according to the findings of some studies, obesity can lead to de novo CKD [7]. The pathophysiology of obesity is sophisticated, including genetic predisposition, environmental changes, and individual preferences. However, about 30% of obese patients seem to be protected against obesity-related metabolic complications [8]. In addition, it has been shown that obesity may have a favourable effect on outcomes and improve survival in chronic diseases such as end-stage kidney disease (ESKD), heart failure, and coronary heart disease. These correlations were first identified in the 1980s, and since then, the phenomenon of the “obesity paradox” has been described by many researchers, especially in haemodialysis patients [9,10,11,12,13].

CKD progresses from its onset and brings many complications. One of them is the systemic disorder chronic kidney disease–mineral and bone disorder (CKD-MBD), which is characterised by either one or a combination of biochemical abnormalities: abnormalities of bone tissue metabolism or vascular or soft tissue calcification [14]. CKD-MBD causes cardiovascular disease with premature arteriosclerosis and accelerated vascular calcification (VC), which leads to generalised VC [15,16,17]. The theoretical protective effect of obesity on VC formation and prognosis of CKD patients is an understudied field. In our previous work, we have shown that obesity does not have a favourable effect in VC in CKD patients, independently of kidney function. Furthermore, VC was more pronounced in obese patients with reduced kidney function [18]. Therefore, we aimed to evaluate the prognostic value of VC on long-term outcomes in obese and non-obese CKD patients.

## 2. Materials and Methods

Patients older than 18 years with different stages of CKD (G1A3-G5A3) were recruited to a single-centre, prospective observational study. The main cohort included 168 consecutive patients from the Nephrology department at Tartu University Hospital. The current study sample consisted of 150 patients from the main cohort. Patients were selected from the main cohort based on the availability of the required data. The study was carried out from January 2012 to the end of March 2024. CKD was defined according to KDIGO guidelines by eGFR and albuminuria [19].

Participation in the study was voluntary and anonymous; all participants provided written informed consent. The Ethics Review Committee on Human Research at the University of Tartu approved the study (approval no. 223/T-17). Baseline demographic, clinical, and biochemical data were collected at the time of enrolment. Basic clinical data were collected: age, gender, aetiology of CKD, and concomitant medication. Physical examination of subjects was performed, including palpation of peripheral pulses of the lower limbs and measurement of blood pressure. Body weight, height, and body mass index (BMI) were measured. Patients were divided into two groups based on their BMI scores—a group with a BMI ≥ 30 kg/m^2^ and a group with a BMI < 30 kg/m^2^.

Laboratory analyses were performed at the United Laboratories of Tartu University Hospital.

Laboratory parameters included serum haemoglobin (S-Hb, g/L), serum creatinine (S-Crea, µmol/L), serum—urea (S-Urea, mmol/L), serum albumin (S-Alb, g/L), serum total calcium (S-Ca, mmol/L) and ionised calcium (S-i-Ca, mmol/L), serum phosphate (S-Pi, mmol/L), serum uric acid (S-UA, mmol/L), serum total cholesterol (S-CHL, mmol/L), and serum triglyceride (S-TG, mmol/L).

For measuring serum total alkaline phosphatase (S-tALP, normal range 35–128 iu/L), a kinetic colorimetric assay was used.

Serum 25(OH)D (S-vit D (25 OH), >50 nmol/L) and intact parathyroid hormone (iPTH, 1.6–6.9 pmol/L) were evaluated via an electrochemiluminescence immunoassay using the Elecsys kit (Roche, Basel, Switzerland).

The Chronic Kidney Disease Epidemiology Collaboration (CKD-EPI) equation was applied to estimate kidney function.

For the assessment of intact fibroblast growth factor 23 (iFGF-23, U/mL), serum was obtained from peripheral venous blood samples, stored at −80 °C, and analysed using the ELISA method. According to the description of the ELISA method, the normal range of iFGF-23 is <114 U/mL.

Albumin-to-creatinine ratio (g/mol) was used to assess albuminuria. Urine samples were collected from first-void urine and analysed by immunoturbidimetric assay.

Vascular calcification was defined as abnormal deposition of calcificates in the arterial wall and heart valves. To assess calcification, we used a lateral lumbar spine X-ray, the ankle–brachial index (ABI), and echocardiography.

### 2.1. Abdominal Aortic Calcification Score

A lateral lumbar spine X-ray film was taken, and abdominal aortic calcification (AAC) was scored by two radiologists who were blinded to the clinical data, using the Kauppila score. The abdominal aorta next to the first four lumbar vertebrae was divided into four segments, using the midpoints of the intervertebral spaces as boundaries. The segments of the anterior and posterior aortic wall were assessed separately. Calcific deposits were graded on a scale of 0–3 at each segment as follows: 0—no calcific deposits; 1—small scattered calcific deposits filling less than one third of the aortic wall; 2—one third to two thirds of the aortic wall calcified; 3—at least two thirds of the aortic wall calcified. A Kauppila calcification score, spanning 0–24 points, was obtained by summing the scores of the eight aortic segments [20]. When conducting the statistical analysis of the long-term outcome, we divided the subjects into two groups: with no calcific deposits (grade 0) and with calcific deposits.

### 2.2. Ankle–Brachial Index (ABI)

For the assessment of ABI, systolic blood pressure (SBP) was measured in the brachial artery of the arms and in the posterior tibial and dorsal pedal arteries at both ankles with the Atys Microflow Doppler ultrasound device. ABI was calculated by the following formula: ABI = ankle SBP/brachial SBP. The worst observed result was chosen to represent the ABI. Patients were assigned to two ABI classifications: ABI ≥ 0.9 to <1.3 in both feet—norm; <0.9 or ≥1.3 in either foot—pathologically low or high ABI, respectively. Low ABI is associated with peripheral arterial disease (PAD), and high ABI is associated with arterial stiffness [21].

### 2.3. Echocardiography

Echocardiography was performed by experienced cardiologists. Left ventricular hypertrophy (LVH) was defined as suggested by the American Society of Echocardiography/European Society of Echocardiography chamber quantification guideline [22]. The calcification and fibrosis of heart valves were evaluated according to standard echocardiography methodology and classified as present or not present.

### 2.4. Statistical Analysis

All analyses were performed using Statistica (version 14.01.25) software. Tests were two-sided, and a *p*-value of <0.05 was considered statistically significant.

Means and standard deviations were calculated for continuous variables, and percentages were calculated for categorical variables.

Survival curves were estimated by the Kaplan–Meier product-limit method and compared by the log-rank test.

### 2.5. Long-Term Outcome

CV complications and mortality were used as end-points. Among CV complications, stroke, myocardial infarction, decompensated heart failure, amputation due to atherosclerosis, and aortic rupture were assessed.

## 3. Results

The mean age of participants (N = 150) was 60 years (SD 14, range 22–88 years), and 69 patients (46%) were males. The aetiology of CKD was hypertension in 41 (27%), diabetes in 39 (26%), glomerulonephritis in 32 (21%), interstitial nephritis in 17 (11%), polycystic kidney disease in 7 (5%), genetic and congenital disorders in 3 (2%), and other in 11 (8%) patients. Fifty-eight patients (39%) were obese with a BMI ≥ 30 kg/m^2^. The results regarding clinical and laboratory data are presented in Table 1.

The results of an abdominal X-ray to measure the Kauppila score were available in 87 patients (42%); in 39 (45%) of them, the score was 0, and in 48 (55%), the score was at least 1. Among patients with visible deposits in the aortic wall, 25 had a moderate calcification score for the abdominal aorta (1–6), and 23 had a severe (7–24) one.

The ABI was normal in 76 patients (51%) out of 149, high (≥1.3) in 45 patients (30%), and low (<0.9) in 28 patients (19%).

Echocardiography was performed in 109 patients. LVH was found in 68 patients, and heart valve lesions (calcinosis and fibrosis) were found in 55 patients.

By the 11.2-year follow-up, 70 patients had died (47%). Twenty-five percent of deaths occurred within the first 7 years of follow-up. Among patients with a BMI ≥ 30 kg/m^2^ (N = 58), 27 patients died. In the group with a BMI < 30 kg/m^2^ (N = 92), 43 patients died.

Twenty-four patients had CV complications: stroke, myocardial infarction, decompensated heart failure, amputation caused by atherosclerosis, or aortic rupture.

**Figure 1:**
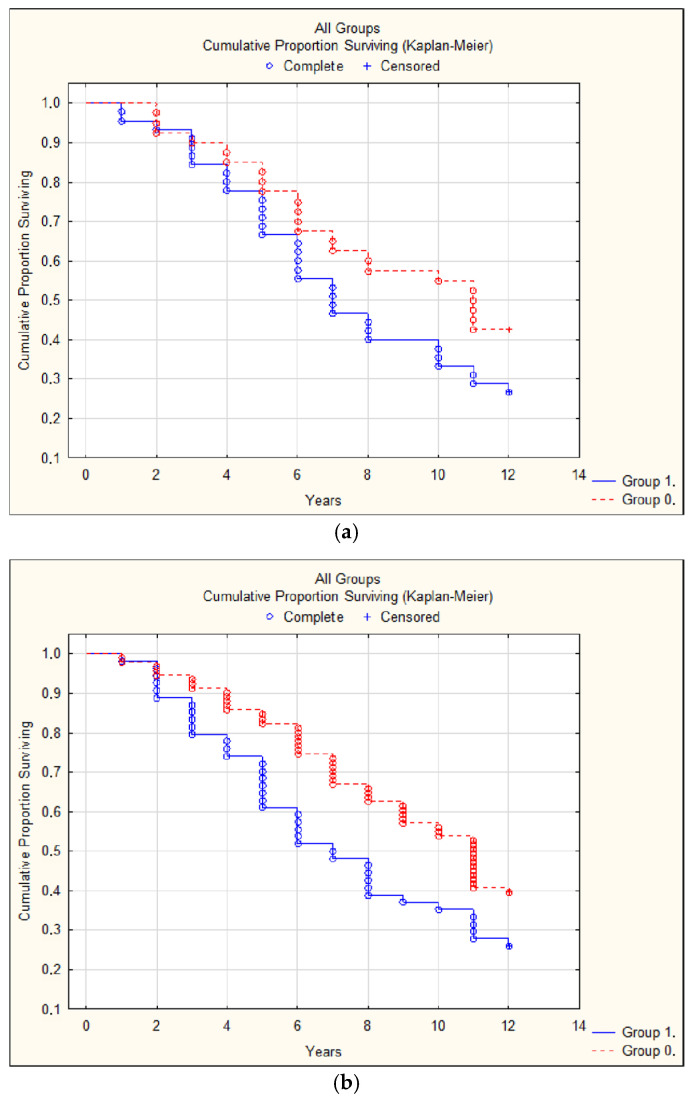
(**a**) Complications (stroke, myocardial infarction, decompensated heart failure, amputation due to atherosclerosis, or aortic rupture) with Kauppila score > 1 (group 1) and Kauppila score 0 (group 0). *p* = 0.04. (**b**) Complications (stroke, myocardial infarction, decompensated heart failure, amputation due to atherosclerosis, or aortic rupture) with ABI ≥ 1.3 or <0.9 (group 1) and normal ABI (group 0). *p* = 0.02.

**Figure 2:**
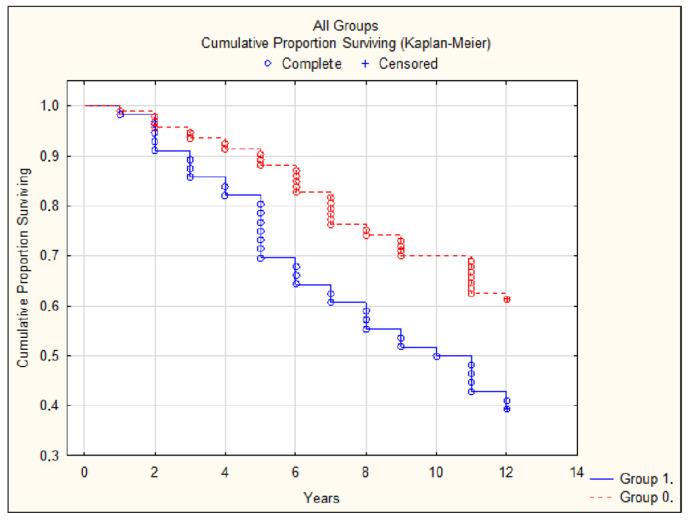
All-cause mortality in group with ABI ≥ 1.3 or <0.9 (group 1) and with normal ABI (group 0) *p* = 0.006.

In the whole group, an AAC score more than 1 (*p* = 0.04) (Figure 1a), a pathologically high or low ABI (≥1.3 or <0.9) (*p* = 0.02) (Figure 1b), the existence of LVH (*p* = 0.001), and heart valve lesions (*p* = 0.03) were statistically significant predictors of CV complications. Moreover, abnormal ABI (*p* = 0.006) (Figure 2), LVH (*p* = 0.009), and valve lesions (*p* = 0.02) were predictors of all-cause mortality.

Among obese patients (BMI ≥ 30 kg/m^2^), only LVH was a significant predictor of CV complications (*p* = 0.01) and mortality (*p* = 0.004). In patients with a BMI < 30 kg/m^2^, predictors of CV complications and mortality were ABI (*p* = 0.03; *p* = 0.009), LVH (just for CV complications, *p* = 0.02), and heart valve lesions (*p* = 0.009; *p* = 0.004).

There were no differences in the measured parameters of VC between the obese and non-obese groups.

**Figure 3:**
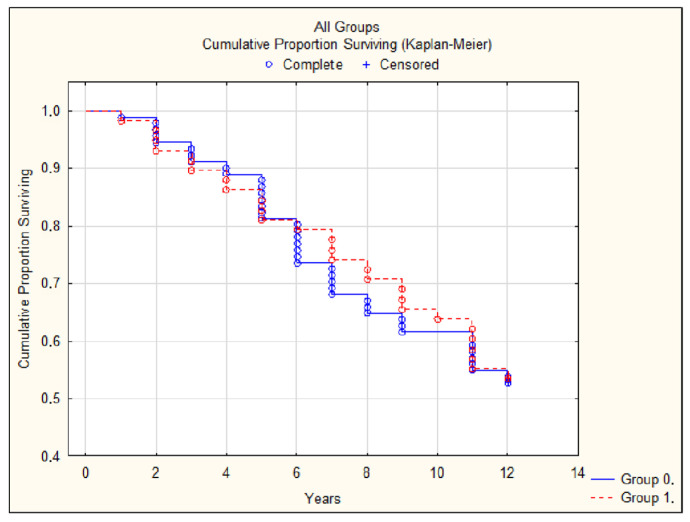
Survival in patients with a BMI < 30 (group 0) and a BMI ≥ 30 (group 1).

**Figure 4:**
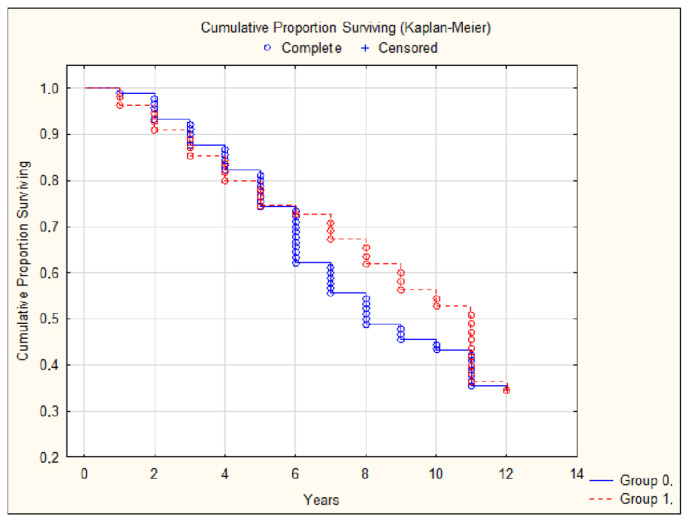
Complications (stroke, myocardial infarction, decompensated heart failure, amputation caused by atherosclerosis, or aortic rupture) in patients with a BMI < 30 (group 0) and a BMI ≥ 30 (group 1).

Moreover, comparing patients with and without obesity according to the studied parameters, we found no significant differences in complications and mortality (Table 2, Figure 3 and Figure 4).

In an unadjusted Cox proportional model, AACS (HR 1.06) and eGFR (HR 0.96) were associated with CV complications; age (HR 0.03) and eGFR (HR 0.97) were associated with mortality (Table 3a,b).

## 4. Discussion

The results of this work showed the associations between markers of VC, complications, and mortality in our CKD cohort. However, obesity did not improve patient outcomes; the results of patients with and without obesity were similar.

Similarly to the results of previous studies, we confirmed the associations of high AAC and an increased risk of CV complications in CKD patients. However, in this work, we did not find associations between AAC and mortality, despite these findings being described by many other authors. The AAC score is independently associated with CV complications and death in the general population and dialysis patients [23,24,25,26,27]. In the KNOW-CKD study, AAC was independently associated with adverse CV outcomes in pre-dialysis patients [28]. AAC is linked to structural heart abnormalities such as LVH and atrial hypertrophy. Altogether, these changes lead to CKD progression and CV complications [29,30]. Some authors have also described the effect of ACC on kidney disease outcomes and CKD progression [31]. Moreover, Lankinen et al. described rapid progression of AAC score in patients with CKD 4–5 on different RRT modalities or conservative treatment [32]. Current guidelines recommend assessing AAC in individuals with CKD [14].

In the current study, we showed that ABI predicts CV complications and mortality in CKD patients. ABI is a simple, non-invasive measure, and its pathologically low value (<0.9) is one sign of subclinical PAD, and its high value (≥1.3) indicates arterial stiffness [33]. Both low and high ABI are associated with an elevated risk of mortality in the general population and in patients with CKD [34,35,36,37]. Furthermore, previous research has demonstrated that abnormally high or low ABI is an independent indicator of CV and all-cause mortality in CKD patients [21,38,39]. Moreover, a retrospective cohort study showed that high ABI independently predicts major adverse CV events [40]. Additionally, low ABI is a powerful predictor of a decline in kidney function [41].

Both valvular calcification and LVH predicted CV complications and mortality in our cohort. Interestingly, LVH was the only significant predictor of outcome in patients with obesity. Valvular calcification is a serious extraosseous calcification in CKD. Heart valve calcification leads to LVH and alteration of cardiac function [42,43]. Our previous study showed a significantly higher presence of LVH in patients with lower kidney function [18], and similar results were described by others [44]. The 5-year mortality rate among patients with at least mild aortic stenosis or mitral regurgitation was more than 50% greater than in individuals without CKD [45].

In the previous study in the same cohort, we demonstrated that patients with high BMI had a higher risk of VC formation, especially those with reduced kidney function; the obesity paradox was not present in this group [17]. The current study in the same cohort showed that obesity does not have a positive impact on long-term outcomes either; the rate of CV complications and mortality was similar in both groups.

Some studies have suggested that overweight and obesity may have a positive impact on patient outcomes [12,13,46,47]. However, consistent with our findings, some studies have reported no differences in mortality between obese and non-obese CKD patients [48]. Thus, the relationship between obesity and mortality, along with its underlying mechanisms, remains complex and not yet fully understood [49].

Additionally, due to the short life expectancy in ESRD patients, the survival benefits observed in obese patients may, in the short term, outweigh the harmful long-term effects of obesity [50]. Additional studies are required to better understand the effects of obesity on VC and its progression and outcomes in CKD patients.

### Strengths and Limitations of This Study

This study is a continuation of our previous research, where we examined the impact of VC on prognosis and complications in obese and non-obese CKD patients, with a long follow-up period.

We used multiple diagnostic methods for effectively assessing VC. Although these are well-known methods, this is still a new approach to assessing VC, which could be implemented more into everyday clinical practice. However, further investigations are needed to optimise the choice of comprehensive research.

Our study also had some limitations. The study cohort was relatively small, and a larger sample may be needed for a better understanding of the associations between obesity and VC in CKD patients to improve CKD patient outcomes.

Although BMI is routinely used for the evaluation and classification of obesity, it is an indirect measure and does not distinguish between adipose and muscle mass.

## 5. Conclusions

VC is a significant complication that negatively impacts the prognosis of patients with CKD. Overall, VC serves as an adverse prognostic marker in CKD, increasing the risk of CV complications and worsening the course of the disease. Obesity does not have a protective effect on long-term outcomes in CKD patients.

## Figures and Tables

**Table 1:** Clinical and laboratory characteristics of the whole group and obese (BMI ≥ 30 kg/m^2^) and non-obese (BMI < 30 kg/m^2^) groups.

	Whole Group	BMI < 30 kg/m^2^	BMI ≥ 30 kg/m^2^	*p*-Value
N	150	92	58	
Male	46%	52%	36%	0.04
Mean age	60 ± 14	57.8 ± 15.7	64.2 ± 12.4	0.02
Body weight (kg)	82.2 ± 19.1	72.0 ± 11.9	98.4 ± 17.0	0.001
Hb (g/L)	124.3 ± 18.4	121.7 ± 18.4	128.5 ± 17.7	0.03
Creatinine (μmol/L)	152.0 (51–1036)	181.5 (54–1015)	136.5 (51–1036)	0.002
Urea (mmol/L)	13.6 ± 7.6	14.3 ± 7.8	12.5 ± 7.1	0.09
eGFR (mL/min/1.73 m^2^)	36.4 ± 24.3	32.5 ± 22.9	42.5 ± 25.3	0.02
Calcium (mmol/L)	2.3 ± 0.2	2.3 ± 0.2	2.3 ± 0.2	0.6
Ionised calcium (mmol/L)	1.3 ± 0.1	1.3 ± 0.2	1.3 ± 0.1	0.4
Phosphate (mmol/L)	1.3 ± 0.5	1.4 ± 0.5	1.3 ± 0.5	0.5
PTH (pmol/L)	10.0 (0–320.4)	10 (0–320.4)	9.5 (2.9–175.9)	0.8
Vitamin D (nmol/L)	52.9 ± 26.3	52.9 ± 29.2	52.9 ± 21.6	0.5
iFGF-23 (U/mL)	43.5 (10–2400)	34 (10–2400)	58.5 (11–2044)	0.02
Albumin (mmol/L)	41.3 ± 5.8	40.7 ± 6.8	42.3 ± 3.6	0.5
CRV (mg/L)	2.0 (1–107)	2.0 (1–107)	2.0 (1–28)	0.8
Uric acid (mmol/L)	396.3 ± 107.4	383.3 ± 106.3	416.5 ± 106.9	0.02
Total cholesterol (mmol/L)	5.5 ± 1.3	5.5 ± 1.2	5.3 ± 1.4	0.2
HDL-cholesterol (mmol/L)	1.5 ± 1.4	1.7 ± 1.8	1.3 ± 0.5	0.01
LDL-cholesterol (mmol/L)	3.5 ± 1.1	3.5 ± 1.1	3.5 ± 1.1	0.8

BMI, body mass index; Hb, haemoglobin; CRV, C-reactive protein. Statistically significant *p* value between the obese and non-obese groups was <0.05.

**Table 2:** Parameters of vascular calcification, CV complications, and mortality in the obese (BMI ≥ 30 kg/m^2^) and non-obese (BMI < 30 kg/m^2^) groups.

	BMI < 30 kg/m^2^	BMI ≥ 30 kg/m^2^	*p*-Value
AACS > 1	27.2%	39.7%	0.1
ABI < 0.9 and ≥1.3	53.3%	43.1%	0.2
LVH, yes/no	40.2%	51.7%	0.2
Valves lesions, yes/no	35.9%	46.6%	0.2
Mortality	47.3%	46.6%	0.9
CV complications	65.6%	65.5%	0.9

CV, cardiovascular; BMI, body mass index; AACS, abdominal aortic calcification score; ABI, ankle–brachial index; LVH, left ventricular hypertrophy.

**Table 3:** (**a**) Associations between abdominal aortic calcification score, kidney function, and CV complications (stroke, myocardial infarction, decompensated heart failure, amputation caused by atherosclerosis, or aortic rupture) in the whole group (unadjusted Cox regression model). (**b**) Associations between age, kidney function, and mortality in the whole group (unadjusted Cox regression model).

Variable	*p*-Value	HR (95%CL)
(a)		
eGFR	0.0004	0.97 (0.95; 0.98)
AACS	0.01	1.06 (1.01; 1.12)
(b)		
Age	0.01	1.03 (1.01; 1.06)
eGFR	0.006	0.97 (0.95; 0.99)

CV, cardiovascular; eGFR, estimated glomerular filtration ratio; AACS, abdominal aortic calcification score; HR, hazard ratio.

## Data Availability

The data that support the findings of this study are available from the corresponding author upon reasonable request.

## Abbreviations

AAC	abdominal aortic calcification
AACS	abdominal aortic calcification score
BMI	body mass index
CKD	chronic kidney disease
CKD-MBD	chronic kidney disease–mineral and bone disorder
CVD	cardiovascular disease
ESKD	end-stage renal disease
LVH	left ventricular hypertrophy
PAD	peripheral arterial disease
SBP	systolic blood pressure
VC	vascular calcification
